# TAFRO Syndrome: A Case Report from Turkey and Review of the Literature

**Published:** 2018-10-01

**Authors:** Zeki Islamoğlu, Ali Erkan Duman, Göktuğ Sirin, Hasan Yılmaz, Meral Uluköylü Mengüç, Yiğit Erçetin, Süheyla Bozkurt, Sadettin Hülagü, Altay Çelebi

**Affiliations:** 1 Department of Gastroenterology, Faculty of Medicine, Kocaeli University, Kocaeli, Turkey; 2 Department of Hematology, Faculty of Medicine, Kocaeli University, Kocaeli, Turkey; 3 Department of Pathology, Faculty of Medicine, Marmara University, Istanbul, Turkey

**Keywords:** TAFRO syndrome

## Abstract

TAFRO syndrome is a rare subtype of the Castleman’s disease which has been described over the last years. The name of TAFRO syndrome comes from thrombocytopenia, anasarca, myelofibrosis, renal dysfunction, and organomegaly. We report a young Turkish male patient presented with fever, night sweats, fatigue, nausea, bilateral pretibial pitting edema, abdominal pain and watery diarrhea. PET/CT revealed multiple lymphadenopathies in cervical, axillary, mediastinal, paraaortic, mesenteric and inguinal lymph nodes. Excisional lymph node biopsy showed atretic germinal centers and expanded interfollicular areas, containing sheets of plasma cells. The R-CHOP regimen was started, and his signs and symptoms improved after the treatment. The current case confirms the unique presentation of this syndrome, helping to understand its clinical course and treatment strategy.

## Introduction

 Castleman’s disease (CD) is a rare benign lymphoproliferative disorder. It represents a form of nonclonal lymph node hyperplasia   ^1^^,^^2^ . Surgical removal of the enlarged node which is also the treatment of the disease in a unicentric form is necessary for the diagnosis^   3 ^. On the basis of the extent of local lymph node involvement, CD classifies as unicentric (UCD) or multicentric Castleman’s disease (MCD). MCD is subdivided into HHV-8-associated MCD and HHV-8-negative or idiopathic MCD (iMCD) based on the presence of Human Herpes Virus-8 (HHV-8).

iMCD is a very rare syndrome. An estimated 1000 new cases are diagnosed with iMCD each year in the United States which is slightly more common in men and older ages^   4 ^. iMCD is associated with an overproduction of IL-6 in a portion of patients, leading to C-reactive protein production in hepatocytes and contributing to the inflammatory syndrome^   5 ^. The etiology of iMCD is not known, and there are no known risk factors for the disease. iMCD has at least three clinical subtypes: POEMS syndrome, (polyradiculoneuropathy, organomegaly, endocrinopathy, monoclonal plasma cell proliferative disorder, and skin changes), TAFRO syndrome (thrombocytopenia, anasarca, myelofibrosis, renal dysfunction, and organomegaly), and iMCD-not otherwise specified (iMCD-NOS)^   6 ^. 

In 2010, Takai et al. reported three iMCD cases presented with high fever, anasarca, hepatosplenomegaly, lymphadenopathy, thrombocytopenia, and reticulin fibrosis of the bone marrow^   7 ^. In 2012, TAFRO syndrome was first described at the Fukushima and Nagoya meetings^   8 ^. To date, over 30 cases were reported, and most of them were from Japan. Here, we report the first case of TAFRO syndrome in Turkey.

## Case presentation

 A 19-year-old male was presented with fever, night sweats, fatigue, nausea, bilateral pretibial pitting (+2) edema, abdominal pain and watery diarrhea for two weeks. Dyspnea and abdominal distention were added to his complaints in the following days. He had no chronic disease history. The physical examination revealed bilateral pulmonary rales accompanied with decreased breath sounds in the lung bases, massive ascites, and mild splenomegaly. In a week time, cervical, axillary, submandibular and inguinal lymphadenopathies were showed up. 

Full blood count showed bicytopenia: platelet count 16 × 10^3^/ µl, hemoglobin 8.64 (HCT 26.14), MCV 73.92 fL. White blood cell number and sedimentation rate were within normal range. Acute kidney injury was demonstrated by increased creatinine (2.38 mg/dl) and blood urea nitrogen (75mg/dl). 70 mg of protein in 24-hour urine collection was detected.  Serum B12 and Fe levels were both decreased: 75 pg/dl (145-505) and 15mg/dl (50-175), respectively. Other laboratory anomalies were: Albumin 2.70 gr/dl (3.5-5.2), ALP 247 U/L (30-120), CRP 20 gr/L (<0.5), prothrombin time (PTZ) 17.1 sec (11.5-15.5), INR 1.4.

Blood and urine cultures which include bacteria and fungi were studied multiple times because of fever over 38 °C, and all the culture results were negative. Serologic and autoantibody tests for Anti-HIV-1,2, CMV, EBV, HSV, Toxoplasmosis, Rubella, HCV, HBV, HAV, Brucellosis, Salmonella, Syphilis were ordered, and they were all negative. Autoimmune markers which include ANA and ENA panel were all negative. Serum levels of immunoglobulins  (IgG, IgA, IgM) were in normal range. IgG4 level was also studied, and it was in normal range [0,12 ug/ml (0,09-0,20)]. In the following days, serum and urine immunofixation tests were also studied because of suspected of POEMS syndrome, and the results were both negative for a monoclonal protein. 

His thyroid function tests were compatible with central hypothyroidism: TSH 0.12 µIU/m (0.38-5.53), fT4 0.32 ng/dl (0.61-1.2). Additionally low testosterone level [0.47 ng/ml (1.75-7.8)] and high ACTH level [168 pg/ml (0-45)] were detected. Prolactin, growth hormone, FSH, LH, and cortisol hormone levels were in normal range.

We performed a paracentesis for the diagnostic and therapeutic purpose. It revealed a high serum-ascites albumin gradient (SAAG) (2.7) and high total protein level (2.6 gr/dl) in ascitic fluid. Analysis of ascitic fluid for cytological and microbiological did not show any significant result. Electromyography (EMG) of upper and lower extremity was requested due to arm and leg cramps with numbness, and the findings were normal.

**Figure 1:**
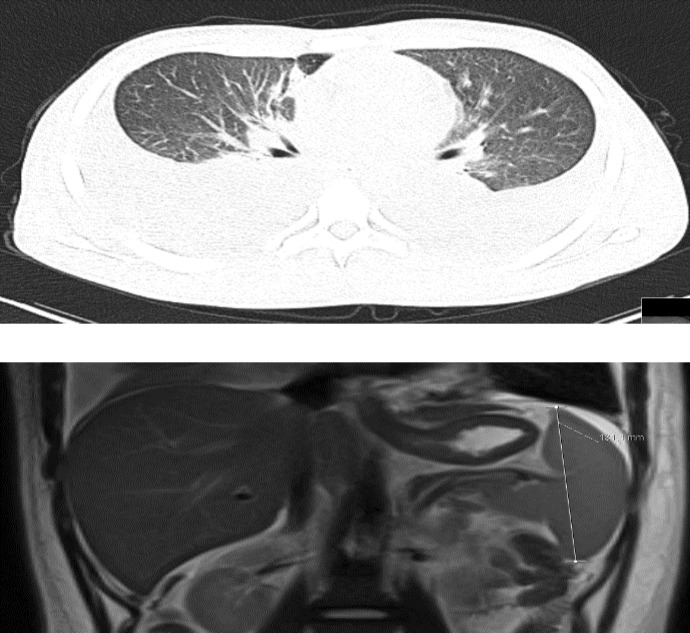
Bilateral parenchymal ground-glass opacities, patchy rounded areas of consolidation, and interlobular septal thickening were detected on CT scan. A mild splenomegaly was shown on MRI scan.

During follow-up, the patient's dyspnea got worse and blood oxygen levels started to fall outside the normal range. A contrast-enhanced computed tomography (CT) was ordered and bilateral parenchymal ground-glass opacities, patchy rounded areas of consolidation and interlobular septal thickening prominent in lower lobe basal segments were detected. Bilateral pleural effusions (5cm on the left, 7cm on the right), multiple enlarged mediastinal and hilar lymph nodes (1 to 2 cm diameter), and a mild splenomegaly were also detected**. **Similarly, abdominal magnetic resonance imaging (MRI) scan showed multiple lymphadenopathies in paraaortic, mesenteric and inguinal lymph nodes with a mild splenomegaly (Figure 1). Bilateral tube thoracostomy was performed after platelet transfusion. Pleural fluid was exudative. The gram stain and culture (both aerobic and anaerobic) of the pleural fluid were studied and the results were both negative. Additionally, pleural fluid examination for tuberculosis (TB), including polymerase chain reaction (PCR), auramine–rhodamine (AR)-stain and mycobacterial culture were also applied. No significant results for TB were detected. 

**Figure 2:**
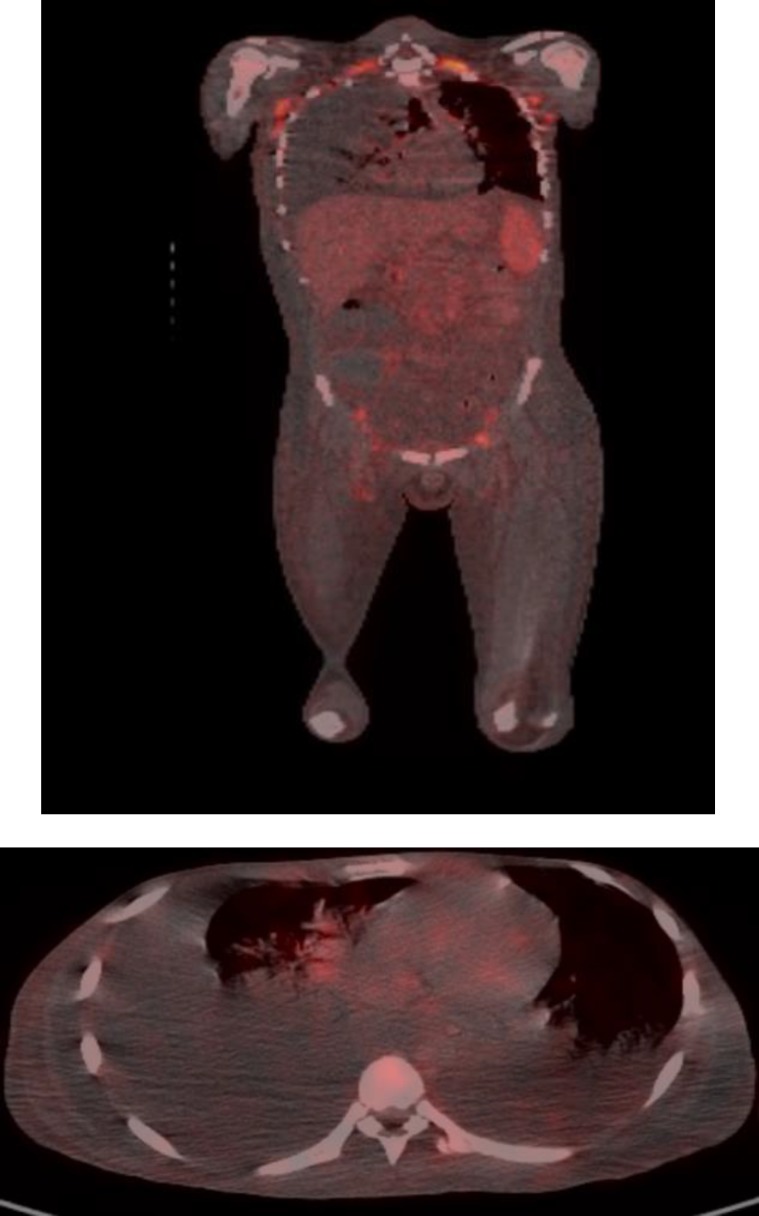
Multiple lymphadenopathies in cervical, axillary, mediastinal, paraaortic, mesenteric and inguinal lymph nodes. FDG uptake rates were from 3.6 to 8.5.

A Positron Emission Tomography/Computed Tomography (PET/CT) was ordered with the suspicion of the lymphoma. It revealed multiple lymphadenopathies in cervical, axillary, mediastinal, paraaortic, mesenteric and inguinal lymph nodes. The diameters of the lymph nodes were 11 to 20 mm, and standardized uptake values (SUVs) were from 3.6 to 8.5 (Figure 2).

**Figure-3:**
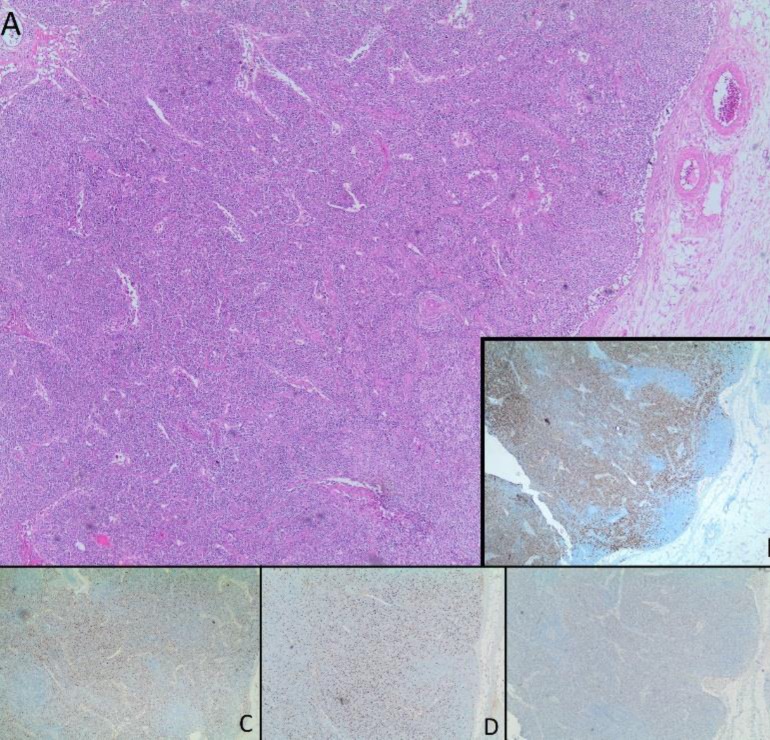
Atretic germinal centers and expanded interfollicular areas containing sheets of plasma cells (Fig. A). Immunohistochemical stain for CD138 highlighting the plasma cells (Fig. B), Immunohistochemical stains for Kappa and Lambda light chains showing polytypic nature of plasma cells (Fig. D and E) and negative HHV-8 staining (Fig. E).

A bone marrow aspiration was performed, and it showed hypocellular marrow with the absence of megakaryocytes. Maturation of the myeloid cells was complete, and blasts rate were <%1. Due to the absence of atypical cells in bone marrow aspiration, we didn't perform bone marrow biopsy. Left axillary lymph node dissection was performed. Postoperative microscopical examination showed atretic germinal centers and expanded interfollicular areas containing sheets of plasma cells. LANA-1 staining for HHV-8 was negative (Figure 3).

**Figure 4:**
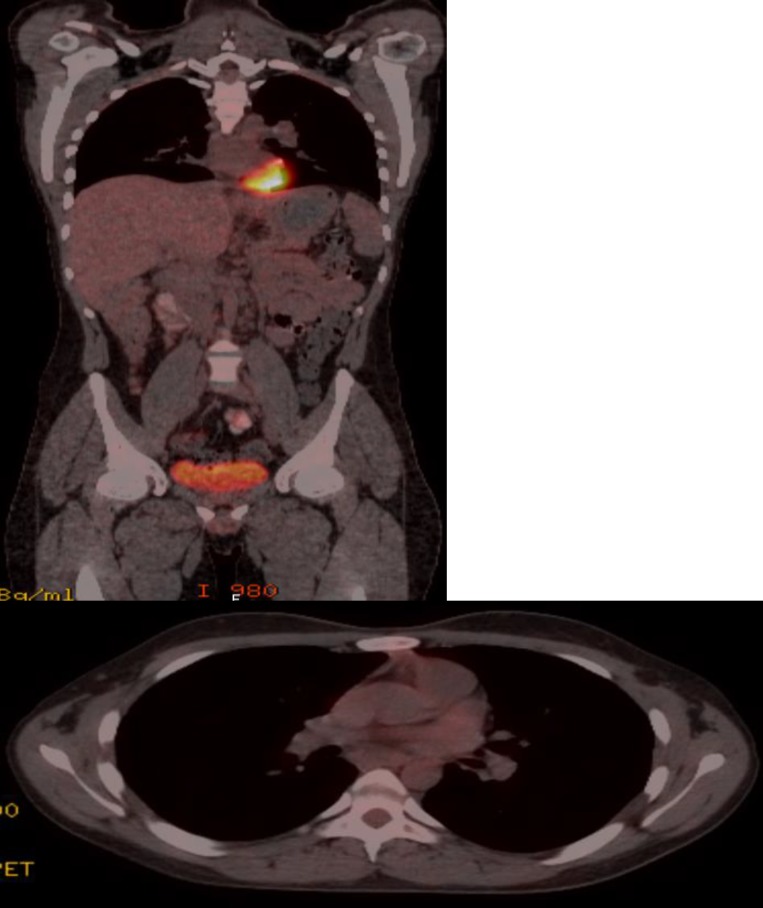
PET/CT showed bilateral a few 1 to 2 cm diameter cervical lymphadenopathies without FDG uptake. It was interpreted as complete metabolic remission.

The patient was diagnosed with TAFRO syndrome. We chose R-CHOP regimen for the treatment because of the absence of the anti-IL-6 therapies in our country and good steroid response which was experienced during follow-up. This regimen was repeated 2 more times at 21-day intervals. Grade 1-2 cytopenias which did not necessitate hospitalization occurred. The patient had a good response to this therapy, and his clinical, laboratory and radiologic findings were improved. After 1 year of diagnosis, the patient underwent physical examination, laboratory and radiological investigations. The patient was in complete remission in all parameters including metabolic remission on PET/CT (Figure 4). However, the patient underwent three more cycles of R-CHOP regimen. 

## Discussion

 TAFRO syndrome is a rare systemic inflammatory disorder which has recently been described and is difficult to diagnose. The defining characteristics include thrombocytopenia (T), anasarca (A), fever (F), reticulin myelofibrosis (R), and organomegaly (O). Whether TAFRO syndrome should be an independent entity or classified as a subvariant of iMCD is still a controversial issue. 

Two papers published in 2016 focused on clinical, pathological and diagnostic criteria of TAFRO syndrome^6^^,^^9^. Iwaki et al. analyzed the clinical features and histopathological characteristics of 25 iMCD cases that demonstrated TAFRO clinical symptoms^   6 ^.They recommended classifying iMCD into TAFRO-iMCD and iMCD-NOS (not otherwise specified).TAFRO-iMCD cases showed more aggressive clinical course, corticosteroid-refractoriness, thrombocytopenia, higher frequency of anasarca, an elevated level of ALP, and normal gammaglobulin levels. They proposed the diagnostic criteria for TAFRO-iMCD which required compatible histopathological findings, three major criteria and one or more minor criteria (Table-1). TAFRO characteristic findings of lymph nodes describe atrophic germinal centers with enlarged nuclei of endothelial cells, the proliferation of endothelial venules with enlarged nuclear in the interfollicular zone, and small numbers of mature plasma cells. Some diseases should also be excluded, including rheumatologic diseases such as SLE, infectious diseases such as acute Epstein - Barr virus, and neoplastic diseases such as lymphoma, POEMS syndrome and cancer. 

**Table 1:** Diagnostic Criteria of Iwaki et al. for TAFRO-iMCD^   6 ^

1. Histopathological Criteria; Compatible with pathological findings of lymph nodes as TAFRO-iMCD• Negative LANA-1 for HHV-8 2. Major criteria; • Presents 3 of 5 TAFRO symptoms: Thrombocytopenia, Anasarca, Fever, Reticulin fibrosis, Organomegaly • Absence of hypergammaglobulinemia • Small volume lymphadenopathy 3. Minor criteria need 1 or more; • Hyper/normoplasia of megakaryocytes in bone Marrow • High levels of serum ALP without markedly elevated serum transaminase

Masaki et al. took a different approach to diagnosis and classification of TAFRO syndrome^   9 ^. They proposed histological features consistent with CD in lymph nodes as a one of the four minor categories. Three major categories (anasarca, thrombocytopenia, and systemic inflammation) and 2 out of 4 minor categories were necessary for the diagnosis (Table-2). A diagnosis of TAFRO syndrome requires excluding malignancies, autoimmune disorders, infectious disorders, POEMS syndrome, IgG4-related disease, hepatic cirrhosis and thrombotic thrombocytopenic purpura (TTP)/hemolytic uremic syndrome (HUS). Based on their diagnostic criteria, patients may be assigned a diagnosis of the TAFRO syndrome without histological characteristics of MCD. Masaki et al. consider TAFRO and iMCD as overlapping syndromes because some of the TAFRO patients do not fulfill the criteria of iMCD. They also propose a disease severity classification for TAFRO syndrome based on the severity of major categories and renal insufficiency. 

**Table 2:** Diagnostic Criteria of Masaki et al. for TAFRO syndrome^   9 ^.

Major categories: 1. Anasarca, including pleural effusion, ascites, and general edema 2. Thrombocytopenia; defined as a pretreatment platelet count ≤ 100,000/μL 3. Systemic inflammation: fever of unknown etiology above 37.5 °C and/or serum CRP ≥ 2 mg/dL * Requirements; all major criteria, and two or more minor criteria	Minor categories: 1. Lymph node biopsy shows Castleman's disease-like histopathologic features 2. Bone marrow shows reticulin myelofibrosis and/or increased number of megakaryocytes 3. Mild organomegaly, including hepatomegaly, splenomegaly, and lymphadenopathy Progressive renal insufficiency

Our case report meets all the major and minor criteria as mentioned Iwaki et al. He also fulfilled all major and minor categories that Masaki et al. described. We excluded all the diseases which are in the differential diagnosis as they mentioned by Masaki et al. The disease severity which was established by Masaki et al. was grade 4 (severe). Lymphadenopathy in TAFRO syndrome is usually smaller than 1.5 cm in diameter, huge lymphadenopathy may indicate lymphoma and other diseases ^9^^,^^10^ . Besides, massive enlargement of bilateral cervical and axillary lymph nodes (>15 mm), mild enlargement of (<15 mm) mediastinal, intraabdominal and pelvic lymph nodes were also detected on PET/CT in our case. There are no sufficient data about the value of FDG PET-CT in diagnosis and follow-up of the TAFRO syndrome. HHV-8-associated MCD and iMCD are both PET avid usually with a relatively low SUV of 2.5-511. SUV rates of the current case varied between 3.6 and 8.5, and mediastinal lymph nodes had the highest SUV rate.

Since the TAFRO syndrome is classified as a subgroup of iMCD, it could show some unique features which can be seen in iMCD cases. In 2017, an international team established first diagnostic criteria for iMCD^   4 ^. Both major criteria and two or more minor criteria (at least one laboratory must be included) are needed for the diagnosis (Table-3). Also, infection-related disorders (HHV-8, EBV-lymphoproliferative disorders, CMV, toxoplasmosis, HIV, active tuberculosis, etc.), autoimmune/autoinflammatory diseases (systemic lupus erythematosus, rheumatoid arthritis, adult-onset Still disease, juvenile idiopathic arthritis, autoimmune lymphoproliferative syndrome) and malignant/lymphoproliferative disorders (lymphoma, multiple myeloma, primary lymph node plasmacytoma, FDC sarcoma, POEMS syndrome) must be excluded. The current case fulfilled 2 major criteria, and 9 of 11 minor criteria (of which 5 were laboratory criteria). We detected bilateral parenchymal ground-glass opacities, patchy rounded areas of consolidation, and interlobular septal thickening on thoracic CT. Also, bilateral pleural effusions, multiple enlarged mediastinal and hilar lymph nodes had been accompanied to parenchymal lesions. Johkoh et al. described that these kinds of pulmonary parenchymal findings are due to the associated lymphocytic interstitial pneumonitis which can be seen in MCD^   12 ^. iMCD patients can exhibit POEMS-like complications without concurrent POEMS syndrome^   4 ^. Central hypothyroidism and primary hypogonadism were detected based on laboratory findings. Moreover, an elevation in ACTH level with normal cortisol level was detected. We accepted this situation as a relative adrenal insufficiency due to severe illness. For clinical deterioration of the patient, oral hydrocortisone treatment followed by L-thyroxine and testosterone was started before the definitive diagnosis. The complaints of the patient including fever, night sweats, dyspnea, fatigue and abdominal pain significantly improved**. **In the following days after the definitive diagnosis of TAFRO syndrome, this early hormonal treatment could suggest that our case had a good steroid response.

**Table 3:** 2017 International Consensus Diagnostic Criteria of iMCD^10^

1. Major Criteria: • Histopathologic lymph node features consistent with the iMCD spectrum • Enlarged lymph nodes (≥1 cm in short-axis diameter) in ≥2 lymph node stations 2. Minor Criteria: Laboratory: • Elevated CRP (>10 mg/L) or ESR (>15 mm/h) • Anemia (hemoglobin <12.5 g/dL for males, hemoglobin <11.5 g/dL for females) • Thrombocytopenia (platelet count <150 k/μL) or thrombocytosis (platelet count >400 k/μL) • Hypoalbuminemia (albumin <3.5 g/dL) • Renal dysfunction (eGFR <60 mL/min/1.73m^2^) or proteinuria (total protein 150 mg/24 h or 10 mg/100 ml) • Polyclonal hypergammaglobulinemia (total γ globulin or immunoglobulin G >1700 mg/dL) Clinical: • Constitutional symptoms: night sweats, fever (>38°C), weight loss, or fatigue • Large spleen and/or liver • Fluid accumulation: edema, anasarca, ascites, or pleural effusion • Eruptive cherry hemangiomatosis or violaceous papules • Lymphocytic interstitial pneumonitis

*Requirements: Both major and two or more minor criteria (at least one laboratory must be included)

Elevation of serum IL-6 level and other inflammatory cytokines have been frequently seen in iMCD^   13 ^. These cytokines induce B-cell and plasma cell proliferation. Vascular endothelial growth factor (VEGF), hypersecretion, acute phase reaction, and angiogenesis are also seen in MCD due to cytokines overload. Iwaki et al. showed in their study IL-6 elevation and related clinical features such as thrombocytosis, polyclonal hypergammaglobulinemia were more commonly seen in iMCD-NOS than TAFRO-iMCD^   6 ^. They suggested that IL-6 may not be the primary pathological cytokine in TAFRO syndrome. 

Masaki et al. proposed a treatment strategy for TAFRO syndrome^   9 ^. High-dose glucocorticoid was suggested as the first-line therapy and Cyclosporin A (CsA) as the second-line therapy for patients refractory to or dependent on glucocorticoids. They also suggested tocilizumab (anti-IL-6 receptor antibody) for patients having TAFRO syndrome complicated by Castleman’s disease, cyclophosphamide combined chemotherapy such as CHOP (cyclophosphamide, doxorubicin, vincristine, and prednisolone) and Rituximab (anti-CD20 antibody). But, these treatment strategies are not evidence-based. Since the etiology and pathology remain unclear, a different therapeutic approach from iMCD is required.  There are limited data on prognosis of TAFRO syndrome. A small group study published by Owattanapanich et al. declared that most CD patients had a good prognosis, whereas TAFRO syndrome had a poor outcome^   14 ^. Three-year overall survival in the TAFRO group was 50%. 

In conclusion, it remains controversial whether TAFRO syndrome should be a subgroup of MCD or constitutes a distinct entity. The treatment of TAFRO syndrome is also controversial. Unlike our case, most of the cases didn’t respond well to the steroids. Since TAFRO Syndrome is recently described, we will have more evidence-based knowledge as new cases are published.
